# Development and psychometric characteristics of the SCI-QOL Pressure Ulcers scale and short form

**DOI:** 10.1179/2045772315Y.0000000017

**Published:** 2015-05

**Authors:** Pamela A. Kisala, David S. Tulsky, Seung W. Choi, Steven C. Kirshblum

**Affiliations:** 1 Department of Physical Therapy, University of Delaware, College of Health Sciences, Newark, DE, USA; 2 Kessler Foundation, West Orange, NJ, USA; 3 CTB/McGraw-Hill, Monterey, CA, USA; 4 Kessler Institute for Rehabilitation, West Orange, NJ, USA; 5 Department of Physical Medicine and Rehabilitation, Rutgers New Jersey Medical School, Newark, NJ, USA

**Keywords:** Health-Related quality of life, Patient reported outcomes, Pressure ulcer, Skin, Spinal cord injuries

## Abstract

**Objective:**

To develop a self-reported measure of the subjective impact of pressure ulcers on health-related quality of life (HRQOL) in individuals with spinal cord injury (SCI) as part of the SCI quality of life (SCI-QOL) measurement system.

**Design:**

Grounded-theory based qualitative item development methods, large-scale item calibration testing, confirmatory factor analysis (CFA), and item response theory-based psychometric analysis.

**Setting:**

Five SCI Model System centers and one Department of Veterans Affairs medical center in the United States.

**Participants:**

Adults with traumatic SCI.

**Main Outcome Measures:**

SCI-QOL Pressure Ulcers scale.

**Results:**

189 individuals with traumatic SCI who experienced a pressure ulcer within the past 7 days completed 30 items related to pressure ulcers. CFA confirmed a unidimensional pool of items. IRT analyses were conducted. A constrained Graded Response Model with a constant slope parameter was used to estimate item thresholds for the 12 retained items.

**Conclusions:**

The 12-item SCI-QOL Pressure Ulcers scale is unique in that it is specifically targeted to individuals with spinal cord injury and at every stage of development has included input from individuals with SCI. Furthermore, use of CFA and IRT methods provide flexibility and precision of measurement. The scale may be administered in its entirety or as a 7-item “short form” and is available for both research and clinical practice.

## Introduction

Pressure ulcers (PrU) in persons with spinal cord injury (SCI) are clearly one of the most devastating secondary complications in terms of effects on individuals' overall quality of life. Not only is PrU development the most common secondary complication all years post injury^1^ but it can also be deadly.^2^ Despite the attention given to preventive strategies during both acute and rehabilitative care, between 50–80%^3–7^ of individuals with SCI develop a PrU at some point after injury. Annual incidence of PrU in SCI has been variously reported as 31–52%, with up to 79% of these individuals experiencing recurrent PrU.^1,8–14^ Finally, diseases of the skin including PrU are the second most common cause of re-hospitalization after SCI,^15^ and 8% of individuals with SCI and PrU will die from PrU-related complications.^2^

Pressure ulcers are defined as an injury to the skin and/or underlying tissue, typically over a bony prominence, as a consequence of pressure extended over a long period of time, or pressure in conjunction with friction and/or shear.^16,17^ Individuals with SCI are particularly susceptible to PrU given the amount of time spent lying or sitting and the lack of sensory feedback relied upon by able-bodied individuals to initiate position changes. Individuals with SCI also exhibit higher sitting pressures than able-bodied adults, likely due to the atrophy of the muscle tissue that typically performs a protective function.^18^ Alterations in skin collagen^19^ and decreased tissue oxygenation^20^ after SCI increase susceptibility to PrU development. Furthermore, shear occurs when the skin remains stationary but the underlying tissue shifts in response to force applied tangentially to the skin's surface. In individuals with SCI, shear is likely to occur during everyday activities such as bathing, dressing, or transferring.^21^

Development of a PrU can have devastating consequences on an individual's health related quality of life (HRQOL). At a minimum, PrU management is time-consuming and inconvenient, requiring frequent weight shifts and/or dressing changes. Furthermore, PrU may be accompanied by an exudate that can stain clothes or have a foul odor and can be a source of pain in individuals with incomplete injuries.^22^ Pressure ulcers can have physical consequences such as limitation of sitting time and thereby engagement in daily activities, emotional consequences such as self-consciousness or embarrassment, and social consequences including reduced intimacy and avoidance of social activities.^23^ Recovery from a PrU or from surgery to correct a PrU often involves prolonged bed rest, which is often accompanied by a loss of productivity, income, and self-worth, as well as a delay in obtaining vocational and rehabilitation goals.^24^ The direct costs associated with PrUs can also create a significant financial burden, with the cost of treating a PrU reaching $30,000–$70,000 exclusive of increased hours of personal assistance and/or long-term skilled nursing care.^14^

Injury-related factors such as level and completeness of injury, longer duration of SCI, and degree of functional independence are considered risk factors for PrU.^1,14,25^ Male sex, use of tobacco and alcohol, and poor nutrition are also associated with PrU development.^26,27^ Race has been associated with PrU development, PrU severity, and necessity of surgical PrU repair.^7,28–30^ Socioeconomically, unemployment and low educational achievement have both been linked with an increased prevalence of PrU.^25^ Several medical conditions, such as diabetes mellitus, and cardiac, pulmonary, and vascular diseases, may contribute to the development of PrU and can cause delays in wound healing.^31^ Finally, having had a PrU in the past is a significant risk factor for developing PrU in the future.^5,14,32^

Many argue that HRQOL can only be assessed from the patient's perspective, and this subjective evaluation is critical to “understanding the cognitive processes that mediate the patient's perceptions (p. 187)” of the impact of their pressure ulcer.^33^ It is necessary to measure a person's perception of their symptoms, impact of the PrU, and extent of disruption of one's life, especially because treatment side effects may outweigh clinical improvement. Unfortunately, despite the prevalence and associated impact of PrUs, there are currently no patient-reported outcome (PRO) measures to assess the subjective impact of PrU on HRQOL in individuals with SCI. One non-SCI specific measure, the Cardiff Wound Impact Schedule (CWIS),^34^ was developed with individuals with lower leg ulceration or diabetic foot ulceration and contains 28 items across the areas of physical, emotional, and social health. However, the included items assess HRQOL generally and are not necessarily attributable to PrUs. Although this scale was developed specifically for individuals with leg ulcerations, it is the only known measure targeting the effect of PrUs on HRQOL. Other researchers have utilized generic measures of health status and life satisfaction in studies examining HRQOL in individuals with PrUs and SCI.^35^ For example, the Medical Outcomes Study Short Form-36^36^ is a health status measure intended for a general health population but also contains items about such diverse areas as physical and emotional functioning. Hitzig *et al*.^35^ also reported that the Life Situation Questionnaire-Revised^37^ and the Ferrans and Powers Quality of Life Index for SCI v3,^38^ two PRO measures of subjective HRQOL have been used in individuals with SCI and pressure ulcers, but neither of these instruments contain items that specifically address PrUs or how skin problems impact HRQOL.

A different category of available outcomes measures assess the risk of developing a PrU (e.g. the Braden Scale^39^ or the SCI-specific Spinal Cord Injury Pressure Ulcer Scale^40^ and Spinal Cord Injury Pressure Ulcer Scale-Acute),^41^ or the extent to which an existing PrU has healed (e.g. the Spinal Cord Impairment Pressure Ulcer Monitoring Tool),^42^ but these are objective measures that do not take subjective perceptions or HRQOL effects into account.

The recently published International Spinal Cord injury Data Sets^43^ include the Skin and Thermoregulation Function Basic Data Set^44^ which outlines several variables that should be collected on all individuals with SCI for documentation of clinical indicators. These variables, while important to document the extent and severity of the pressure ulcer, (e.g. location and depth of PrU) do not measure the patient's subjective experience of the PrU, nor the impact on one's HRQOL. There are currently no available PRO measures that incorporate items about PrU. However, individuals with SCI frequently cite PrU and related morbidity^45^ as having substantial negative effects on HRQOL. Given the likelihood that one will experience a PrU following SCI, the severe effect a PrU has on one's physical health and emotional and social functioning, and the qualitative feedback from individuals with SCI about the importance of PrU morbidity, the lack of a PRO to assess the subjective impact of PrU was seen as a significant gap in the assessment of PRO in individuals with SCI. For this reason, the research team prioritized the development of a new item bank to assess the subjective impact of PrU. This paper details the four phases of research that encompassed development and calibration of the Spinal Cord Injury – Quality of Life (SCI-QOL) Pressure Ulcers (PrU) scale.

## Methods

**Figure 1:**
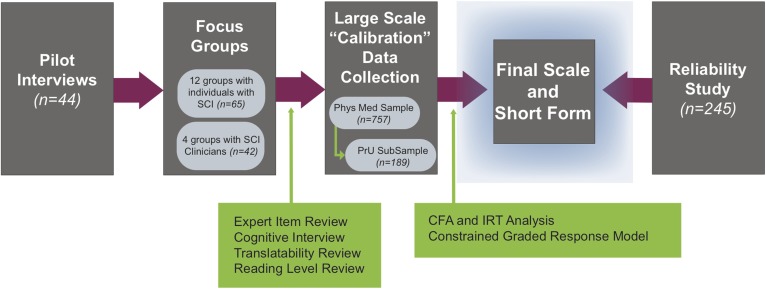
SCI-QOL flow diagram.

The first phase of the SCI-QOL project was to develop items that would comprise a valid and psychometrically sound measure of PrU. Individual interviews and focus groups were used to identify the most important aspects of HRQOL for individuals with SCI, the most relevant aspects of PrU, important HRQOL implications of PrU, and other key items to assess in a skin/pressure ulcers item bank. Preliminary items underwent extensive review and revision through expert item review, cognitive debriefing interviews, translatability review, and reading level review. Final item pools were then tested in large (>700) “calibration” samples of individuals with SCI. Confirmatory factor analysis (CFA) and item response theory (IRT) analysis were used to select and calibrate final items for inclusion in the scale. Finally, test-retest reliability was assessed in a separate sample of individuals with SCI. A flow diagram of the study stages is presented in Fig. 1.

### Item development and refinement

First, a series of 44 semi-structured pilot interviews^46^ was conducted with individuals with traumatic SCI to generate a preliminary list of issues specific to individuals with SCI that impact HRQOL. Notably, participants raised several issues related to pressure ulcers (e.g. *“Health-related aspects of SCI are imperative! Without your health, nothing else matters. Be aware of …skin breakdowns…”)* which highlighted areas for further investigation and formed the initial basis of PRO items.

Next, to garner additional stakeholder feedback on HRQOL, we conducted a series of focus groups with individuals with SCI and a separate set of groups with SCI clinicians.^45^

Across the four participating SCI Model Systems (SCIMS) centers, a total of 12 focus groups were conducted with individuals with SCI (12 groups, *N* = 65), four of which specifically emphasized secondary complications and physical medical issues that individuals with SCI experience. We also conducted a series of four focus groups with SCI clinicians (*N* = 42). Qualitative data from the stakeholder focus groups indicated that 9% of consumer comments and 10% of clinician comments were related to PrU. Based on review of the focus group feedback, study team members drafted 52 items that formed the preliminary SCI-QOL skin/pressure ulcers item pool.

Once the initial item pool was developed, all preliminary items were subject to a rigorous qualitative item review (QIR) process as outlined by the PROMIS^47^ and Neuro-QOL^48^ project teams. The investigative team reviewed the PrU item pool for redundancy, conciseness and content relevance to PrU. During this phase, one item was reworded (“It was a hassle remembering to do my weight shifts/pressure relief” became “I was bothered by having to do weight shifts/pressure relief”) and a total of 21 items were removed. Five of the removed items did not adequately represent the construct (e.g. “I had to sleep on a special mattress to avoid pressure ulcers”), three were too narrow to be useful (e.g. “I had flap surgery for a pressure ulcer”), and 13 items were redundant (e.g. multiple items related to the fear of getting a pressure ulcer). To remain consistent with other SCI-QOL item banks, the context for all of the PrU items would be “In the past 7 days”.

Once the item pool was revised, individuals with SCI (*n* = 5 per item) were called upon to complete, review, and discuss their reaction and understanding of the items in a series of cognitive debriefing interviews.^49^ Five items were modified (e.g. “Pressure ulcers prevented me from working a full [8-hour] day” became “Pressure ulcers prevented me from working my usual number of hours (including work at home)” and two items were removed due to redundancy. An additional four items were added by the study team to address gaps in content coverage that emerged during the cognitive interviewing phase of the project (i.e. items related to the odor, drainage, and dressings for pressure ulcers). The items were also reviewed by a team of language experts to assess translatability potential and flag any words or phrases that would be problematic to translate. During this translatability and cultural review, one item was deemed problematic to translate and was removed. Reading level review was conducted using the Lexile Framework^50^; all items were written at or below a 5^th^-grade reading level. The project team removed 2 additional redundant items during final review, and the resulting pool 30 items were prepared for calibration field testing.

### Item calibration

#### Participants and procedures

A diverse sample of adults with traumatic SCI was recruited from five SCIMS centers and one Department of Veterans Affairs (VA) medical center. The balanced sample was stratified by diagnosis (paraplegia vs. tetraplegia), severity (complete vs. incomplete), and time since injury (<1 year, 1–3 years, and >3 years). SCI-QOL items were administered in interview format by trained examiners, and this study was approved by the institutional review board at each collaborating site. Each participant's diagnosis was confirmed by medical records and each participant's neurologic level was documented by their most recent American Spinal Injury Association Impairment Scale (AIS) rating.^51^ The preliminary pool of 30 PrU items was administered along with other SCI-QOL items related to physical-medical health. Trained data collectors utilized a custom web-based data capture system to read items and record responses, either in person or over the phone. The use of interview format was selected to maximize inclusion of individuals with the widest range of SCI severity and to reduce or eliminate missing data. The data collection procedure is described in more detail in Tulsky *et al.*^52^

#### Analysis

Confirmatory Factor Analytic methods using MPlus version 6.0^a^ were used to assess dimensionality. A variety of fit indices were examined: (1) Tucker-Lewis Index (TLI), a non-normed comparative fit index based on the χ^2^ index divided by degrees of freedom (χ^2^/*df*),^53^ (2) Comparative Fit Index (CFI),^54^ which also compares the current model to a null model, and (3) root mean square error of approximation (RMSEA) which estimates the model discrepancy per degree of freedom.^55^ The commonly accepted criteria of TLI and CFI > 0.9 for good fit and > 0.95 for excellent fit^56^ and RMSEA < 0.08 for acceptable fit^55^ and <0.05 for excellent fit^57^ were used. Item loadings on the single PrU factor were also examined, with a retention criterion of *R*^2^ > 0.3.^58^ As a final step to prepare the items for IRT analysis it was necessary to assess the local item dependence (LID) of the included items.^59^ For any item pair with a residual correlation >|0.2|, one of the items was removed from the item pool. CFA were run iteratively following the removal of each item or group of items.

Once we removed poorly fitting items and verified that the item pool was essentially unidimensional, the graded response model (GRM)^60^ was used to estimate item slope (discrimination) and threshold (difficulty) parameters. As discussed below, final analyses were conducted on a relatively small sample (*n* = 189) and as such it was necessary to use a constrained GRM with a common slope parameter to estimate final thresholds. Then, item fit was further evaluated with the S-X^2^ test using the IRTFIT^61^ macro program, with P < 0.05 indicating poor fit and P < 0.01 necessitating item removal. As with CFA, IRT analyses were re-run in an iterative fashion following removal of any item.

### Short Form Selection and Assessment Center^SM,^^62^ Programming

To select items for a short fixed-length form (as an alternative to the full-scale administration), project investigators (SK, TDH, DT, PK) reviewed the parameters for each item. Since all PrU items were calibrated with a single constant slope, only the thresholds (i.e. item difficulty or “location”) were examined. Items were divided into quintiles based on location, and at each quintile, the 1–2 item(s) with the most face validity and clinical relevance were chosen. To ensure diversity of items in the final form, item wording and similarity to other included items were also considered.

Using the graded response model-estimated IRT parameters, the department of Medical Social Sciences at Northwestern University programmed the PrU scale and the 7-item PrU short form into the Assessment Center^SM^ platform. A thorough quality assurance procedure using test cases and audit trails was used to confirm accuracy of item parameters.

### Reliability study

Two hundred forty-five community-dwelling adults with traumatic SCI who were greater than 4 months post injury at baseline have participated in follow-up study evaluating the reliability, validity and responsiveness of the SCI-QOL. Participants who have been enrolled through 4 collaborating SCIMS centers (University of Michigan, Kessler Institute for Rehabilitation / Kessler Foundation, Rehabilitation Institute of Chicago, and Craig Hospital) complete the 7-item S-PrU short form at baseline, 1–2 weeks, 3 months, and 6 months. All items are administered in interview format by a trained interviewer. The Assessment Center^SM^ platform is used for item administration and data capture. Data from the baseline and 1–2 week retest assessments, respectively, are used to calculate Pearson's *r* and the intraclass correlation coefficient (ICC) to assess test-retest reliability.

## Results

### Item calibration

#### Participant demographic characteristics – individual interviews

A total of 44 community-dwelling individuals with traumatic SCI, including 48% with tetraplegia and 52% with paraplegia participated in the semi-structured individual interviews. Of the sample, 73% were male. Fifty-five percent of the sample self-reported as Caucasian, 32% as Black or African-American, 7% Hispanic, and 7% Asian/Pacific Islander.

#### Participant demographic characteristics – focus groups

Demographic information on the 65 individuals with SCI and 42 SCI clinicians who participated in the focus groups is summarized in Tulsky *et al*. (2011)^45^ and is not repeated here.

#### Participant demographic characteristics – calibration study

A total of 757 individuals with traumatic SCI completed the initial interview session containing items related to Physical-Medical health. When analyzing the PrU items, however, the overall sample was found to be highly skewed by the number of individuals who did not endorse having a pressure ulcer in the past 7 days. Consequently, these individuals were removed from further analyses, leaving a final PrU calibration sample of 189 individuals. Among the 189 participants whose data were used to develop the final calibrations, the average age of participants was 42.8 years (SD 15.2). 84% of participants were male and 16% were female. In terms of race, 64% were Caucasian, 22% Black or African-American, 2% American Indian or Alaska Native, 2% more than one race, and 10% were another race or chose not to report their racial background. Additionally, 16% of participants were of Hispanic or Latino origin or descent. Forty-four percent of participants had a high school education or less, while 36% of participants attended some college and 20% of participants completed a Bachelor's degree or higher. Thirty percent of participants were within the first year post-injury, 20% were between 1–3 years post injury, and 50% were greater than 3 years post injury. Mean years since injury were 7.7 (SD 10.5). Forty-two percent of the sample was diagnosed with paraplegia and 58% with tetraplegia. Sixty-seven percent sustained complete injuries while 34% were incomplete. Half (50%) of participants reported using a manual wheelchair for mobility and 62% reported using a power wheelchair (20% reported using both). Six percent were able to ambulate at least some of the time. Additional detail on calibration participant demographics may be found in Table 1.

**Table 1:** SCI-QOL calibration sample demographics

Variable	Physical-Medical domain sample	Pressure Ulcers subsample
	(*n* = 757)	(*n* = 189)
Age	42.9 ± 15.5	42.8 ± 15.2
Sex		
Male	79.1%	83.6%
Female	20.9%	16.4%
Ethnicity		
Hispanic	10.6%	15.9%
Non-Hispanic or Not reported	87.8%	84.1%
Race		
Caucasian	71.1%	64.0%
Black or African-American	17.2%	22.2%
Asian	1.5%	0.5%
American Indian/Alaska native or native Hawaiian/Pacific Islander	0.9%	2.1%
More than one race	1.5%	2.1%
Other or Not reported	6.7%	9.0%
Time since injury (years)	6.7 ± 9.9	7.7 ± 10.5
<1 year post injury	28.9%	30%
1–3 years post injury	27.6%	20%
>3 years post injury	43.5%	50%
Diagnosis		
Paraplegia complete	23.9%	29.1%
Paraplegia incomplete	18.5%	12.8%
Tetraplegia complete	23.1%	37.4%
Tetraplegia incomplete	34.4%	20.7%
Education level		
High school or less	38.4%	44.6%
Some college	33.5%	35.6%
Bachelor's degree or higher	28.1%	19.6%
Injury etiology		
Motor vehicle accident	32.4%	33.9%
Fall	22.3%	18.0%
Gunshot wound/violence	11.8%	13.2%
Diving	6.6%	7.9%
Other sports	7.4%	6.3%
Medical/surgical accident	3.7%	3.2%
Motorcycle accident	2.6%	4.8%
Other or Not reported	6.2%	12.7%
Method(s) of mobility (not mutually exclusive)		
Manual wheelchair	54.4%	50.3%
Power wheelchair	44.1%	61.9%
Ambulation	32.7%	6.3%

#### Participant demographic characteristics – reliability study

Finally, demographic information on the 245 participants in the ongoing reliability study is presented in the introductory paper to this issue.^46^

#### Analysis

Initially, the full sample of 757 individuals was included in the analysis. However, it became clear during the first iteration of analyses that the sample was bimodal, with the majority of participants not having experienced a pressure ulcer in the past 7 days. Inclusion of all 757 individuals in the IRT analyses therefore led to highly skewed data and overly inflated slope estimates. To address this issue, one item (“In the past 7 days … I had a pressure ulcer”) was removed from the pool and used as a screener item, and data from all individuals who responded “Never” to this item were removed at this time. The final analyses reported herein were conducted with data only from the remaining 189 individuals.

We then moved forward with the analytical process, paring down the pool of included items through a 5-step iterative process. Throughout the five iterations of CFA and IRT analyses, a total of 18 items were removed for the following reasons (some items were removed for more than one reason): bimodal distribution (2 items), LID (9 items), sparse cells/collapsed categories (3 items), unacceptable slope value (excessively high slope indicating potential LID, 2 items; low slope, 2 items), and use as a screener item (1 item). The results reported below are based on the final 12-item set.

Among the 12 final items, alpha = 0.924 and item-total correlations ranged from 0.56 to 0.80. All of the items had more than 24% of the sample selecting the first category (“Never” or “Not at all”). No items had sparse data (fewer than 5 observations) in any category, and no items displayed a category inversion (i.e. if a higher score on an individual item corresponded to a lower overall score).

CFA analyses were conducted to examine fit to a unidimensional model. The commonly accepted criteria of CFI and TLI >0.9 for good fit and >0.95 for excellent fit,^56^ and root mean square error of approximation (RMSEA) <0.08 for acceptable fit^55^ and <0.05 for excellent fit^57^ were used. However, RMSEA is sensitive to small sample size^56^ and was therefore expected to be higher than desired. For the final CFA iteration, CFI was 0.961, TLI was 0.952, and RMSEA was 0.124. All items demonstrated acceptable factor loadings, with *R*^2^ for all items exceeding 0.4. One item pair (rSkin3 and rSkin27) did exhibit LID with a residual correlation >|0.2|, with a value of –0.258.

A constrained GRM with a common slope parameter of 2.17 was used to estimate IRT parameters and assess model fit. Final items and parameters are located in Table 2. Threshold values for the 12 items range from −0.84 to 2.02. Measurement precision in the theta range of 0.7 to 1.8 is roughly equivalent to a classical reliability of 0.90 or better. The S-X^2^ test indicated adequate or better model fit for all but one item (rSkin17) at P < 0.05, and for all items and P < 0.01. Marginal reliability was equal to 0.897. Due to the limited sample size (*n* = 189), DIF analyses could not be performed.

**Table 2:** SCI-QOL Pressure Ulcers scale items and IRT parameters

			Item response theory calibration statistics				
Item ID	Response set*	Item stem	Slope	Threshold 1	Threshold 2	Threshold 3	Threshold 4
rSkin3	A	My skin was tender from a pressure ulcer	2.16682	−0.73812	−0.14797	0.28612	0.89255
rSkin4	A	I was frustrated by my pressure ulcer	2.16682	−0.50113	−0.17676	0.28312	0.81460
**rSkin8**	**A**	**A pressure ulcer decreased the quality of my life**	**2.16682**	**−0.19785**	**0.24214**	**0.66984**	**1.13083**
**rSkin9**	**A**	**I had discomfort from pressure ulcers**	**2.16682**	**−0.21646**	**0.14553**	**0.90300**	**1.29272**
**rSkin11**	**A**	**Recovering from a pressure ulcer limited my activities**	**2.16682**	**−0.43421**	**0.00166**	**0.62921**	**1.17471**
rSkin14	A	I was bothered by drainage from a pressure ulcer	2.16682	0.46694	0.93818	1.48129	1.88708
**rSkin17**	**B**	**I spent a lot of time taking care of a pressure ulcer**	**2.16682**	**−0.84524**	**−0.19151**	**0.43848**	**0.98085**
**rSkin27**	**B**	**I was bedridden due to a pressure ulcer**	**2.16682**	**0.26087**	**0.64347**	**1.05071**	**1.39943**
rSkin28	B	A problem with my skin limited my ability to do things	2.16682	−0.07968	0.41119	1.15588	1.45752
rSkin_Com8	B	Pressure ulcers prevented me from working my usual number of hours (include work at home)	2.16682	0.42536	0.71768	1.24076	1.79733
**rSkin_Com15**	**B**	**Pressure ulcers interfered with my social life**	**2.16682**	**0.19655**	**0.55112**	**1.28070**	**1.95893**
**rSkin_Com18**	**A**	**Pressure ulcers interfered with my ability to work**	**2.16682**	**0.63093**	**0.93158**	**1.53047**	**2.01506**

*Context for all items was: ‘In the past 7 days’. Response set A was: Not at all/A little bit/Somewhat/Quite a bit/Very much. Response set B was: Never/Rarely/Sometimes/Often/Always.

**Bold Font** indicates the items selected for the short form.

The PrU scale and short form also contain a non-scored screener item, rSkin18 “I had a pressure ulcer”(Response Set B).

Items and parameters copyright © 2015 David Tulsky and Kessler Foundation. All Rights Reserved. Scales should be accessed and used through the corresponding author or http://www.assessmentcenter.net. Do not modify items without permission from the copyright holder.

Internal consistency reliability for 12-item scale (*α* = 0.927) and 7-item short form (*α* = 0.874) was assessed using Cronbach's alpha.

### Short form selection and assessment center^SM,^^62^ programming

Final item selections resulted in a 7-item short form (SF) entitled “SCI-QOL v1.0 Pressure Ulcers SF7a”. Short form items are indicated with bold text in Table 3. Both the full scale and short form utilize a screener item, rSkin18 “*In the past 7 days … I had a pressure ulcer*”. Individuals who respond “*Never*” to this item should not proceed with the remaining items. The PrU scale may be administered in its entirety or as the 7-item short form. Using the calibration data, the correlation between the full scale and SF administration is 0.96. Additional short forms may be developed in the future and will also be located on Assessment Center^SM^. Assessment Center^SM^ also contains a prototype computer adaptive test (CAT); however, it is considered experimental at this time given the sample and calibration limitations and as such is not available for public use.

**Table 3:** SCI-QOL Pressure Ulcers scale: descriptive item statistics

Item ID	Response Set*	Item Stem	Mean	SD	% at Min	% at Max
rSkin3	A	My skin was tender from a pressure ulcer	2.87	1.549	28.0	23.3
rSkin4	A	I was frustrated by my pressure ulcer	2.86	1.654	36.0	26.5
**rSkin8**	**A**	**A pressure ulcer decreased the quality of my life**	**2.45**	**1.596**	**45.5**	**19.6**
**rSkin9**	**A**	**I had discomfort from pressure ulcers**	**2.38**	**1.489**	**44.4**	**15.3**
**rSkin11**	**A**	**Recovering from a pressure ulcer limited my activities**	**2.61**	**1.549**	**38.1**	**19.0**
rSkin14	A	I was bothered by drainage from a pressure ulcer	1.73	1.206	65.6	6.3
**rSkin17**	**B**	**I spent a lot of time taking care of a pressure ulcer**	**2.91**	**1.479**	**24.3**	**21.7**
**rSkin27**	**B**	**I was bedridden due to a pressure ulcer**	**2.07**	**1.500**	**58.7**	**14.3**
rSkin28	B	A problem with my skin limited my ability to do things	2.19	1.446	49.7	13.8
rSkin_Com8	B	Pressure ulcers prevented me from working my usual number of hours (include work at home)	1.91	1.370	63.3	8.8
**rSkin_Com15**	**B**	**Pressure ulcers interfered with my social life**	**2.03**	**1.339**	**55.8**	**7.5**
**rSkin_Com18**	**A**	**Pressure ulcers interfered with my ability to work**	**1.71**	**1.226**	**69.2**	**6.2**

*Context for all items was: ‘In the past 7 days’. Response set A was: Not at all/A little bit/Somewhat/Quite a bit/Very much. Response set B was: Never/Rarely/Sometimes/Often/Always.

**Bold font** indicates the items selected for the short form.

The PrU scale and short form also contain a non-scored screener item, rSkin18 “I had a pressure ulcer”(Response Set B).

Items and parameters copyright © 2015 David Tulsky and Kessler Foundation. All Rights Reserved. Scales should be accessed and used through the corresponding author or http://www.assessmentcenter.net. Do not modify items without permission from the copyright holder.

**Figure 2:**
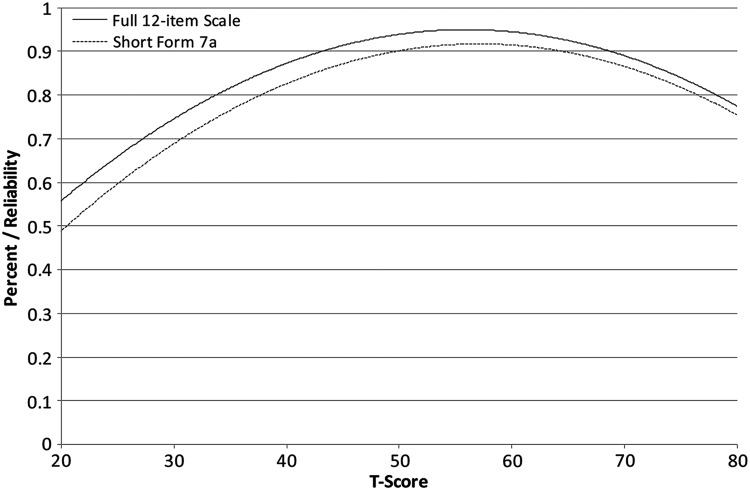
Reliability curves for SCI-QOL pressure ulcers 12-item scale and 7-item short form.

Reliability curves for the full-scale and short-form administrations of the PrU items are located in Fig. 2.

#### Scoring

IRT-based scaled scores were developed and transformed into standard scores on the T metric (mean 50, SD 10). Higher scores indicate greater symptom severity. Given the reference population of individuals with traumatic SCI who have experienced a PrU in the past 7 days, an individual's score indicates the degree to which PrUs are problematic for them compared to other individuals with both SCI and a PrU. A score should be produced for any individual who has responded at a higher level than “1-Never” to the screener item and who has responded to all included items. Scores are produced by summing the raw scores on each of the included items (12 for the full scale and 7 for the short form). Note the response to the screener item is not to be included in score calculations. The raw score can then be converted to a scaled score (on the T metric with a mean of 50 and an SD of 10) using the corresponding lookup tables. Lookup tables are available for the full scale (see Table 4) and 7-item short form (see Table 5). Information on the breadth of coverage for the SF and full scale may be found in Table 6.

**Table 4:** SCI-QOL Pressure Ulcers full scale lookup table

Raw score*	T-score	Standard error	Raw score	T-score	Standard error
12	34.5	5.0	37	58.4	2.4
13	39.0	3.8	38	58.6	2.4
14	39.8	3.6	39	58.8	2.4
15	41.0	3.4	40	58.9	2.5
16	42.1	3.3	41	59.1	2.5
17	43.5	3.1	42	59.4	2.5
18	44.7	3.0	43	59.9	2.5
19	45.9	2.8	44	60.5	2.5
20	47.5	2.7	45	61.2	2.5
21	48.0	2.6	46	62.0	2.5
22	48.7	2.6	47	62.7	2.6
23	49.4	2.6	48	62.9	2.6
24	50.0	2.6	49	63.2	2.6
25	50.5	2.5	50	65.0	2.6
26	51.0	2.5	51	65.2	2.7
27	51.6	2.5	52	65.6	2.7
28	52.2	2.5	53	67.1	2.8
29	52.8	2.4	54	67.3	2.8
30	53.5	2.4	55	67.6	2.9
31	54.5	2.4	56	67.9	3.0
32	55.5	2.4	57	71.2	3.1
33	56.3	2.3	58	71.8	3.2
34	57.1	2.3	59	72.0	3.3
35	57.7	2.3	60	74.4	4.5
36	58.3	2.3			

*Scores are only produced for individuals who respond affirmatively (i.e. >1) to the screener item (rSkin18) and complete all items in the scale (rSkin3, rSkin4, rSkin8, rSkin9, rSkin11, rSkin14, rSkin17, rSkin27, rSkin18, rSkin_Com8, rSkin_Com15, rSkin_Com18). Note that the screener item response is *not* included in the raw/sum score.

**Table 5:** SCI-QOL Pressure Ulcers short form 7a lookup table

Raw score	T-score	Standard error
7	36.7	5.4
8	41.7	4.1
9	43.4	3.9
10	44.7	3.7
11	45.7	3.6
12	47.4	3.5
13	49.4	3.4
14	50.2	3.4
15	52.4	3.3
16	52.5	3.3
17	52.5	3.2
18	54.0	3.2
19	54.6	3.2
20	57.0	3.2
21	57.7	3.1
22	58.5	3.1
23	59.3	3.0
24	60.1	3.0
25	60.9	3.1
26	61.7	3.1
27	62.5	3.2
28	63.3	3.2
29	64.0	3.3
30	64.8	3.3
31	65.8	3.4
32	67.0	3.4
33	68.2	3.4
34	69.7	3.8
35	73.2	4.9

*Scores are only produced for individuals who respond affirmatively (i.e. >1) to the screener item (rSkin18) and complete all items in the short form (rSkin8, rSkin9, rSkin11, rSkin17, rSkin27, rSkin_Com15, rSkin_Com18). Note that the screener item response is *not* included in the raw/sum score.

**Table 6:** Breadth of coverage for 7-item short form and full scale

Mode	T Score				Standard error	
	Mean ± SD	Range	% Floor	% Ceiling	Mean ± SD	Range
7-item short form	51.38 ± 8.86	36.70–73.20	8.90%	0.69%	0.362 ± 0.064	0.300–0.540
Full scale	49.99 ± 9.51	32.94–72.78	4.23%	0.53%	0.305 ± 0.083	0.205–0.543

### Retest reliability study

Test-retest reliability for the 7-item PrU short form was assessed using data from the ongoing reliability study. Participants were included in the analysis if they indicated that they had a PrU “in the past 7 days” at either the baseline and/or the retest assessment. When correlating PrU scores at baseline with those from the 1–2 week follow up assessment (*n* = 245), Pearson's *r* = 0.79 and ICC (2,1) = 0.79 (95% CI: 0.74 to 0.84).

## Discussion

PrUs are a serious secondary medical complication of SCI that are often not only debilitating but can be emotionally, socially, and financially devastating as well. Unfortunately, until now there has not been any way to assess the HRQOL effects of PrUs on individuals with SCI. With the prevalence of PrU prevention regimens and the necessity of medical or surgical intervention for many individuals with PrU, clinicians and clinical trials researchers would greatly benefit from an instrument to assess not only the clinically measurable change in PrU development or healing but also to assess the effects that the prevention or treatment is having on the individual as a whole. The SCI-QOL PrU scale addresses this significant unmet need in its measurement of a range of difficulties associated with PrUs, such as the extent to which PrUs hinder engagement in social, emotional, physical, and recreational activities. The final scale covers topics raised in the literature (e.g. pain, interference with social activities) as well as issues that have been raised specifically by study participants (e.g. drainage). The PrU scale was developed directly from multiple levels of comments and feedback from individuals with SCI and is therefore conceptually grounded to subjectively important PrU-related HRQOL issues.

As with all SCI-QOL item banks and calibrated scales, the PrU scale was developed in adherence to the PROMIS^63^ and Neuro-QOL^64^ methodologies to the full extent possible and therefore extends these measurement systems in a targeted way into the SCI population. The excellent CFI and TLI values also serve as an indicator of the validity of the PrU scale. The PrU scale enables brief, precise measurement of the patient reported HRQOL effects of living with a PrU. This unique measurement tool can be a valuable asset to clinicians and researchers interested in assessing patients' responses to PrU prevention regimens and treatment protocols. The SCI-QOL PrU Scale will be an asset to clinicians in their assessment of PrUs and their impact in individuals with SCI and may be used in conjunction with clinical evaluation and objective measures (e.g. SCIPUS).

### Study limitations

A limitation of the SCI-QOL calibration study is the low rate at which PrU items were endorsed, with only 25% of the sample reporting a pressure ulcer in the past 7 days. This low rate of endorsement initially resulted in highly skewed data and artificially inflated slope parameters. Subsequent analyses were conducted only with data on the select group of individuals who experienced PrU symptoms. Furthermore, while the analytical plan included differential item functioning (DIF) analyses for six categories (age (≤49 vs. ≥50), sex (male vs. female), education (some college and lower vs. college degree and above), diagnosis (tetraplegia vs. paraplegia), severity (incomplete vs. complete), and time post injury (>1 year vs. <1 year), the small sample size prevented us from conducting these analyses.

We have chosen to refer to the final set of 12 PrU items as a “calibrated scale” rather than an item bank given the limited sample size used for calibration, the use of a constrained GRM in threshold estimation, and the small number of retained items. Additional data gleaned from current efforts to assess validity and responsiveness of the PrU Scale and SF will be useful in reassessing the psychometric properties (and nomenclature) of the scale.

## Conclusions

The SCI-QOL PrU scale is a psychometrically sound measurement tool, which can reliably estimate HRQOL effects of PrUs in a traumatic SCI population. The PrU scale and short form are readily available for use in both research and clinical settings. The validation and responsiveness study currently underway will yield data to further evaluate reliability, validity, and sensitivity to change over time. The use of repeated administrations and collection of data on “anchor items” will enable calculation of minimally important differences on the SCI-QOL PrU scale, SF, and CAT. Finally, a notable future direction would be to retest the PrU scale in a sample of at least 500 individuals with SCI who are currently experiencing a pressure ulcer to facilitate recalibration with an unconstrained GRM.

## Suppliers

*Mplus Statistical Analysis with Latent Variables User's Guide* [computer program]. Version 6. Los Angeles: Muthen & Muthen; 2007.

## Disclaimer statements

**Contributors** All authors have contributed significantly to the design, analysis and writing of this manuscript. The contents represent original work and have not been published elsewhere.

**Funding** This study was supported by grant #5R01HD054659 from the National Institutes of Health – Eunice Kennedy Shriver National Institute of Child Health and Human Development/National Center on Medical Rehabilitation Research and the National Institute on Neurological Disorders and Stroke.

**Conflicts of interest** No commercial party having a direct financial interest in the results of the research supporting this article has or will confer a benefit upon the authors or upon any organization with which the authors are associated. All SCI-QOL items and parameters are copyright © 2015 David Tulsky and Kessler Foundation. All rights reserved. All items are freely available to the public via the Assessment Center platform (http://www.assessmentcenter.net). There are currently no plans for Dr Tulsky or Kessler Foundation to benefit financially from the copyright.

**Ethics approval** Institutional IRB approval was received at each participating site.
